# Co-Expression Network Modeling Identifies Specific Inflammation and Neurological Disease-Related Genes mRNA Modules in Mood Disorder

**DOI:** 10.3389/fgene.2022.865015

**Published:** 2022-03-21

**Authors:** Chunxia Yang, Kun Zhang, Aixia Zhang, Ning Sun, Zhifen Liu, Kerang Zhang

**Affiliations:** ^1^ Department of Psychiatry, First Hospital of Shanxi Medical University, Taiyuan, China; ^2^ Shanxi Medical University, Taiyuan, China; ^3^ Nuring College of Shanxi Medical University, Taiyuan, China

**Keywords:** mood disorder, co-expression network analysis (WGCNA), hub genes, inflammation, neurological

## Abstract

**Objectives:** Mood disorders are a kind of serious mental illness, although their molecular factors involved in the pathophysiology remain unknown. One approach to examine the molecular basis of mood disorders is co-expression network analysis (WGCNA), which is expected to further divide the set of differentially expressed genes into subgroups (i.e., modules) in a more (biologically) meaningful way, fascinating the downstream enrichment analysis. The aim of our study was to identify hub genes in modules in mood disorders by using WGCNA.

**Methods:** Microarray data for expression values of 4,311,721 mRNA in peripheral blood mononuclear cells drawn from 21 MDD, 8 BD, and 24 HC individuals were obtained from GEO (GSE39653); data for genes with expression in the bottom third for 80% or more of the samples were removed. Then, the top 70% most variable genes/probs were selected for WGCNA: 27,884 probes representing 21,840 genes; correlation between module genes and mood disorder (MDD+BD vs. HC) was evaluated.

**Results:** About 52% of 27,765 genes were found to form 50 co-expression modules with sizes 42–3070. Among the 50 modules, the eigengenes of two modules were significantly correlated with mood disorder (*p* < 0.05). The saddlebrown module was found in one of the meta-modules in the network of the 50 eigengenes along with mood disorder, 6 (IER5, NFKBIZ, CITED2, TNF, SERTAD1, ADM) out of 12 differentially expressed genes identified in Savitz et al. were found in the saddlebrown module.

**Conclusions:** We found a significant overlap for 6 hub genes (ADM, CITED2, IER5, NFKBIZ, SERTAD1, TNF) with similar co-expression and dysregulation patterns associated with mood disorder. Overall, our findings support other reports on molecular-level immune dysfunction in mood disorder and provide novel insights into the pathophysiology of mood disorder.

## Introduction

Mood disorders including major depressive disorder (MDD) and bipolar disorder (BD) are a kind of serious mental illness and are the third leading cause of the global disease burden (Collins et al., 2011; Murray et al., 2012; Jabbi et al., 2020). The molecular factors involved in the pathophysiology of MDD remain challenging (Gagne et al., 2020). Despite their diagnostic distinction, multiple approaches have shown considerable sharing of risk factors across the mood disorders (Coleman et al., 2020). Various hypotheses regarding the pathogenesis of mood disorders, such as the hypothesis of disturbed neuroplasticity (Christoffel et al., 2011) and the inflammatory (Leonard and Maes, 2012; Zeng et al., 2019), have been proposed. Many studies suggested that neural immune activation may be a primary pathway influencing the observed changes in key neuroendocrine and neurotrophic systems (Miller et al., 2013). Substantial evidence supports the changes in mRNA expression in proinflammatory genes and the elevated levels of peripheral inflammatory markers in mood disorder patients (Kohler et al., 2018; Wiedlocha et al., 2018). However, there is no definitive evidence to support the belief of shared inflammation and neurological abnormalities of molecular biology in mood disorders (Savitz et al., 2013).

Mood disorders share several genetic associations, and can be combined effectively to increase variant discovery (Coleman et al., 2020). Several genome-wide association studies (GWAS) in MDD and BD have indicated that the genetic heterogeneity architecture of mood disorder is complex, with many polymorphisms of small effect contributing to the clinical phenotype (Okbay et al., 2016; Ciobanu et al., 2018; Wray et al., 2018). A recent meta-analysis was conducted using results from the Psychiatric Genomics Consortium (PGC) genome-wide association studies for MDD and BD using data including those from 23andMe and UK Biobank to identify numerous shared and disorder-specific associations between mood disorders. In addition, clinical heterogeneity has been recognized as a major limiting factor for robust characterization of gene expression alterations in MDD. For example, the first RNA sequencing study of 463 lifetime MDD cases, consisting of a mixture of individuals with current and remitted MDD, found no differentially expressed genes between cases and controls (Mostafavi et al., 2014). For BD, neuroimaging-guided RNA-sequencing in two studies showed gene-expression changes associated with disease morbidity and related suicide mortality in an independent postmortem cohort (Jabbi et al., 2020).

To elucidate the relationship between inflammation and neuroimaging abnormalities, Savitz et al. conducted a whole genome expression analysis of peripheral blood mononuclear cells and identified 12 differentially expressed genes including TNF and others that related to neurological disorders and/or apoptosis between patients with a mood disorder and healthy controls. There was mounting evidence that was associated with functional and chemical abnormalities within and beyond the neural reward circuitry and was linked to elevated peripheral levels of inflammatory biomarkers in depression (Ely et al., 2021). An Ingenuity Pathway Analysis on these differentially expressed genes yielded two gene networks centered around TNF and related to cell circle and kinase anomalies, respectively. The authors also found that the expression levels of some of these differentially expressed genes were significantly correlated with morphometric abnormalities of the left sgACC, hippocampus, and caudate. However, there are some limitations in a traditional pathway analysis using IPA, for example. One of the limitations is that the gene networks and regulatory indicated in these networks are modeled based only on currently available knowledge. To fully utilize the gene expression information captured by the microarray data, in this study, we conducted a co-expression network analysis for the microarray data generated in and downloaded from GSE using the WGCNA approach, which was a systems biology approach developed for creating gene network models to explore and identify key functional modules and hub genes.

As far as molecular biology is concerned, genes do not act in isolation. In mood disorder, genes interaction within each other with complex networks might be disrupted. At the same time, gene expression data do not function in isolation but rather are highly multidimensional with complex non-linear biological processes. Molecular interactions are not captured by traditional statistical methods (Ciobanu et al., 2020). Weighted gene co-expression network analysis (WGCNA) is a hypothesis-free systems biology approach that identifies “modules” of co-regulated, and therefore functionally related, genes in a given phenotype (Langfelder and Horvath, 2008), extending classic bivariate approaches (Ciobanu et al., 2018). WGCNA: A systems biology approach developed for analysis of transcriptomic data, providing more information than a set of differentially expressed genes. Used sophisticated algorithms and information on correlation patterns among genes, WGCNA is expected to further divide the set of differentially expressed genes into subgroups (i.e., modules) in a more biologically meaningful way, fascinating the downstream enrichment analysis (Wang et al., 2017).

In this study, we aimed to investigate the relationship between global gene co-expression profiles and mood disorder subgroups. Microarray data for expression values of 4,311,721 mRNA in peripheral blood mononuclear cells drawn from 21 MDD, 8 BD, and 24 HC individuals were obtained from GEO (GSE39653). We applied WGCNA and explored the correlation of co-expressed modules *1*) construct a gene-gene similarity network; *2*) divide the network into modules (group genes with similar expressions); *3*) correlate traits to gene modules; and *4*) identify hub genes in modules. We then sought molecular-level immune dysfunction in mood disorder and provide novel insights into the pathophysiology of mood disorder.

## Methods

### mRNA Microarray Data Acquisition

Microarray data of GSE39653 was downloaded from the National Center for Biotechnology Information (NCBI) Gene Expression Omnibus (GEO, http://www.ncbi.nlm.org/geo/) database, which includes expression levels of 4,311,721 mRNA in peripheral blood mononuclear cells drawn from 21 MDD, 8 BD, and 24 HC individuals. Details on the recruitment of subjects, sampling and processing of the blood samples, and microarray experiment were given in. Briefly, the mood disorder patients met DSM-IV criteria for recurrent primary MDD in a current major depressive episode or BD in a current major depressive episode with a moderate-to-high Hamilton Depression Rating Scale score and did not receive any psychotropic medications for at least 3 weeks. The healthy control individuals had no personal or family history of psychiatric illness. Quantile normalization and log-transformation were performed for the expression data.

### Construction of Weighted Gene Co-Expression Network

First, the microarray data were preprocessed as follows. Data for genes with expression in the bottom third for 80% or more of the samples were removed (Ballouz et al., 2015). The top 70% most variable genes/probs were selected for the construction of the co-expressed network. Using the preprocessed and transformed data, a co-expressed network was constructed using the WGCNA R package (Zhang and Horvath, 2005). Briefly, a correlation matrix for all pair-wise correlations of transcripts was calculated and then transformed into a weighted adjacency matrix with a soft threshold power set to beta = 5 to achieve approximate scale-free topology (model fit R^2 > 0.88 while the mean connectivity was kept as large as possible). The connection strengths were then used to calculate the topological overlap (TO), which is a pair-wise measure of two genes’ similarity with other genes in the network. Genes were then hierarchically clustered using 1-TO as the distance measure and modules of genes were identified using a dynamic tree-cutting algorithm using the following parameters: minimum modulesize = 30, deepSplit = 4, mergeCutSize = 0.15, and maximumBlockSize = 5000.

### Quantification of Module–Trait Associations

The first principal component of each module defined the module eigengene (ME). Genes weakly corrected with the ME (Pearson correlation coefficient<0.3) were removed from the module. For each gene, Pearson correlation coefficient was calculated with the eigengenes of all modules and defined as the model membership (kME). If a gene had the highest correlation and with correlation *p* < 0.05 with the eigengene of a module other than the module it was assigned to initially in the hierarchical clustering, it would be reassigned to this module. Associations between mood disorder (MDD or BD) and MEs were determined by Pearson correlations. Finally, MEs along with the traits were clustered based on their correlation, and the meta-modules were identified to represent groups of correlated modules and/or the traits, which was the affection status of mood disorder in this case.

### Identification of Hub Genes, Functional Annotation, and Gene Ontology Analysis

For modules, significantly associated with mood disorder, the top hub genes were identified as those with the highest intramodular connectivity K_IM, representing the highly connected genes within a module. Functional annotation was performed for those hub genes using the Database for Annotation, Visualization, and Integrated Discovery (DAVID). Gene Ontology (GO) analysis was then performed using the function hyperGTest in the R package Gostats.

### Functional Annotation and Enrichment Analysis

We drew a histogram by mapping the GO (Ashburner et al., 2000; Tweedie et al., 2009) function of genes in modules of interest to the corresponding secondary features. The Pearson Chi-Square test was applied to indicate significant relationships between the two input datasets if all the expected counts were greater than. The top five annotation clusters for each analysis were focused on as these clusters are more likely to contain biologically meaningful annotations as these clusters have the highest enrichment score. Then we implemented GO enrichment analysis based on a hypergeometric test. The *p*-value <0.05 was used as the enrichment cut-off criterion.

## Results

### Data Preprocessing

After removing genes with expression in the bottom third for 80% or more of the samples, the top 70% most variable probes (27,884, representing 21,840 genes) for 29 patients with mood disorder (21 MDD and 8 BD) and 24 HC were selected for WGCNA.

### WGCNA Analysis

**FIGURE 1:**
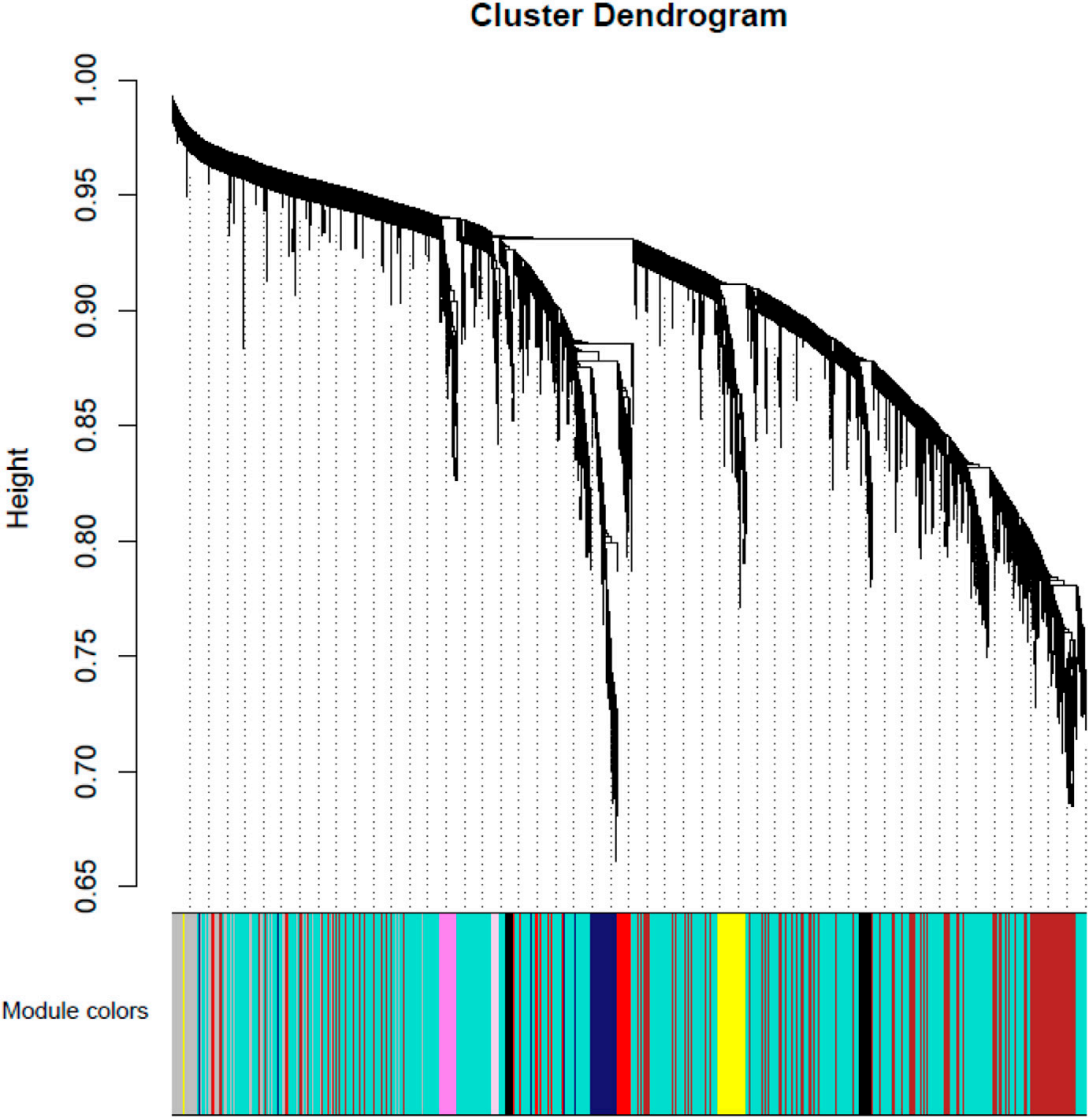
Module-MD vs. Control relationship. Each row corresponds to a module eigengene. Each cell contains the corresponding correlation in the first line and the *p*-value in the second line. The table is color-coded by correlation according to the color legend. Among them, red represents a positive correlation and blue represents a negative correlation.

Weighted gene co-expression networks construction and gene modules identification. Using the preprocessed data for all 53 participants, a weighted co-expression network was constructed using the WGCNA package. The hierarchical clustering procedure and the dynamic tree-cutting algorithm resulted in 50 modules (Figure 1 and Supplementary Table S1), each of which is assigned a unique color label and visualized in the color band underneath the cluster tree in Figure 1. These modules ranged in size from 42 genes in the thistle2 module to 3070 in the turquoise module. Among all 27,765 probes, 13,240 (47.7%) were found to belong to none of the 50 proper modules and were put in an improper module (gray).

### Co-Expression Modules Correlated With Mood Disorder

**FIGURE 2:**
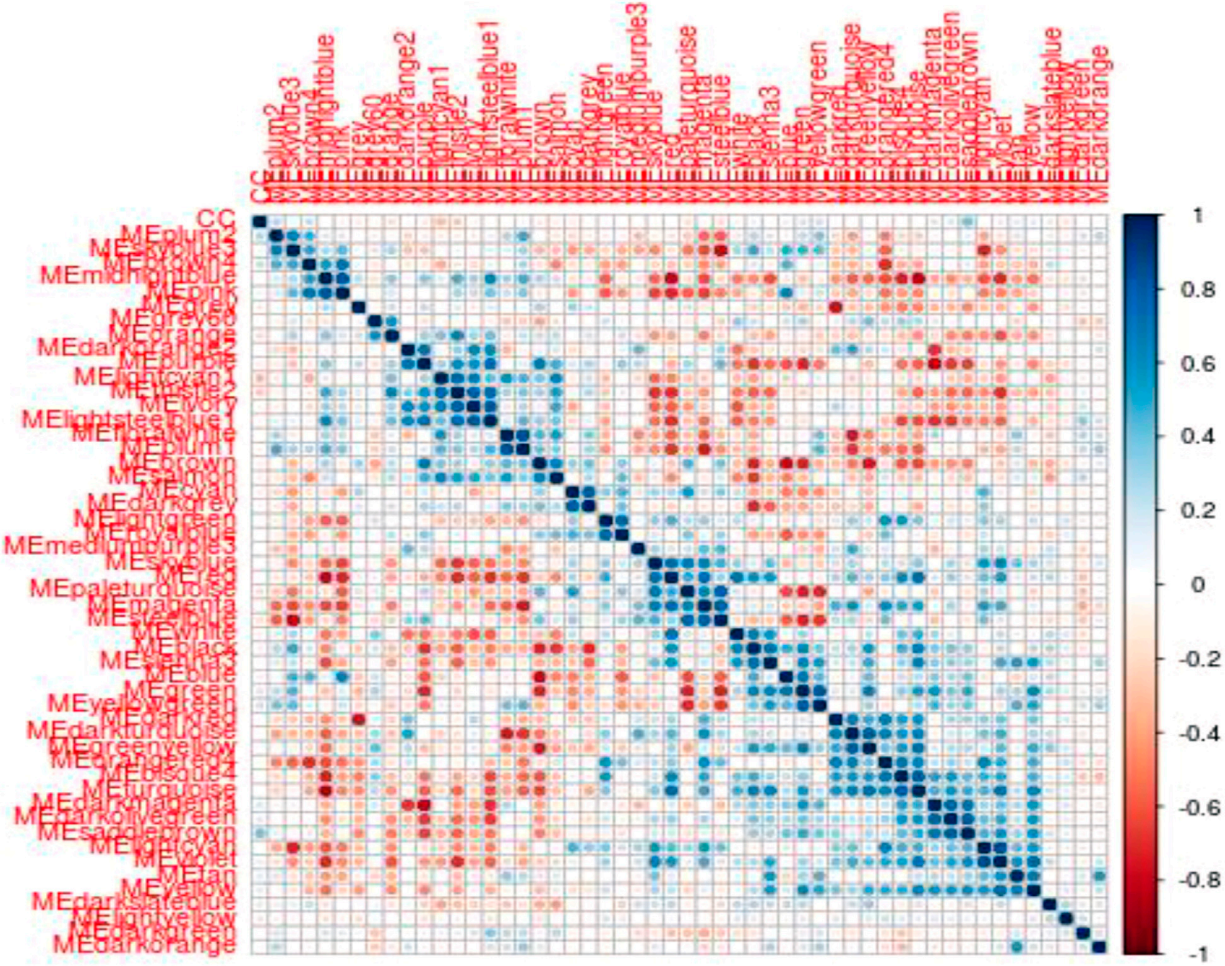
Eigengene heatmap: The scatterplots of MM vs. GS in saddlebrown module exhibited very significant positive correlations.

To identify modules related to mood disorder, we correlated each of the 50 module eigengenes with the mood disorder status. As shown in Table 1, the saddlebrown module and the lightcyan module were significantly associated with mood disorder. The saddlebrown module, which was positively associated with mood disorder, included 10 out of the 26 genes that were found in Coleman et al. (2020) to be differentially expressed between patients with mood disorder and heathy controls. Among these 10 genes, the following 6 are protein coding genes: ADM, CITED2, IER5, NFKBIZ, SERTAD1, and TNF, which were mostly related to neurological features or psychiatric illness. The lightcyan module was negatively associated with mood disorder, and did not include any of the 26 differentially expressed genes identified in Coleman et al. (2020). This implies that the lightcyan module might represent some pathway which may not be identified using differential expression analysis of individual genes. It should be noted that 6 coding genes in the saddlebrown module that were differentially expressed are all over-expressed in the mood disorder cases. The saddlebrown module was also found in the same meta-module in the network of the 50 eigengenes along with mood disorder, as shown in Figure 2. In the same meta-module, there were two more eigengenes representing the darkmagenta module and the darkolivegreen module.

**TABLE 1:** Two candidate modules speculated the critical role for the pathophysiology of MD.

Module	Spearman_CC (*p*-value)	Module	Spearman_CC (*p*-value)
MEmediumpurple3	−0.012 (0.9)	MElightcyan	0.017 (0.9)
MEskyblue	0.045 (0.8)	MEviolet	0.15 (0.3)
MEpaleturquoise	−0.2 (0.1)	MEgrey60	−0.059 (0.7)
MEmagenta	−0.077 (0.6)	MEorange	−0.21 (0.1)
MEsteelblue	−0.21 (0.1)	MEfloralwhite	−0.027 (0.8)
MElightgreen	0.052 (0.7)	MEplum1	0.097 (0.5)
MEroyalblue	−0.042 (0.8)	MEbrown	−0.15 (0.3)
MEcyan	−0.13 (0.3)	MEpurple	−0.15 (0.3)
MEdarkgrey	−0.087 (0.5)	MEsalmon	−0.14 (0.3)
MEdarkred	0.037 (0.8)	MEivory	−0.11 (0.4)
MEorangered4	−0.082 (0.6)	MElightsteelblue1	−0.042 (0.8)
MEdarkorange2	−0.059 (0.7)	MElightcyan1	−0.33 (0.02)
MEdarkturquoise	−0.03 (0.8)	MEthistle2	−0.28 (0.05)
MEgreenyellow	0.079 (0.6)	MEdarkgreen	0.047 (0.7)
MEbisque4	−0.045 (0.8)	MEdarkslateblue	0.17 (0.2)
MEturquoise	0.025 (0.9)	MElightyellow	−0.17 (0.2)
MEred	−0.02 (0.9)	MEdarkorange	0.079 (0.6)
MEwhite	−0.12 (0.4)	MEplum2	0.28 (0.04)
MEblack	0.12 (0.4)	MEbrown4	0.23 (0.1)
MEsienna3	0.089 (0.5)	MEmidnightblue	0.037 (0.8)
MEdarkmagenta	0.18 (0.2)	MEpink	0.11 (0.4)
MEdarkolivegreen	0.18 (0.2)	MEskyblue3	0.079 (0.6)
MEsaddlebrown	0.42 (0.002)	MEblue	0.14 (0.3)
MEtan	0.16 (0.2)	MEgreen	0.2 (0.1)
MEyellow	0.17 (0.2)	MEyellowgreen	0.22 (0.1)
		MEgrey	−0.15 (0.3)

### Identifying Hub Genes From Candidate Modules

Hub genes for the saddlebrown and lightcyan1 modules were extracted and ranked based on the intramodular connectivity values (Wang et al., 2017). Thus, we identified the hub genes from the saddlebrown module (Table 1 for the saddlebrown module). Among the 12 genes identified in Savitz et al. (2013) as differentially expressed between controls and cases (mood disorder), 6 appeared in the saddlebrown module (Supplementary Table S2).

### Functional Annotation of Mood Disorder Correlated Modules and GO Analysis

Hub genes for the saddlebrown and lightcyan1 modules were extracted and ranked based on the intramodular connectivity values. The top hub genes were annotated using the GeneCard website. Most of the hub genes in the saddlebrown module were related to the similar function of RNA processing, in which mRNA 5′-splice site recognited, mRNA cis spliced *via* spliceosome, and branching involved in labyrinthine layer morphogenesis. Moreover, most of the hub genes in the lightcyan1 module were related to the similar function of regulating steroid hormone secretion. GO analysis for the hub genes of these two modules suggested the genes were enriched in the regulation of corticosteroid hormone secretion. Interestingly, 10 of the 19 GO terms pathway in these two modules were associated with the steroid hormone secretion pathway, including negative regulation of steroid hormone secretion (GO:2000832, *p* = 2.29E−05) and regulation of corticosteroid hormone secretion (GO:2000846 *p* = 6.31E−04) (Table 2).

**TABLE 2:** Functional annotation of modules and network analysis.

Module	GOBPID	*p* value	OddsRatio	ExpCount	Count	Size	Term
lightcyan1	GO:2000832	2.29E−05	0	0.01	2	2	Negative regulation of steroid hormone secretion
lightcyan1	GO:2000847	2.29E−05	0	0.01	2	2	Negative regulation of corticosteroid hormone secretion
lightcyan1	GO:2000850	2.29E−05	0	0.01	2	2	Negative regulation of glucocorticoid secretion
lightcyan1	GO:0035933	2.27E−04	142.69	0.024	2	5	Glucocorticoid secretion
lightcyan1	GO:2000849	2.27E−04	142.69	0.024	2	5	Regulation of glucocorticoid secretion
lightcyan1	GO:0035929	6.31E−04	71.33	0.039	2	8	Steroid hormone secretion
lightcyan1	GO:0035930	6.31E−04	71.33	0.039	2	8	Corticosteroid hormone secretion
lightcyan1	GO:2000831	6.31E−04	71.33	0.039	2	8	Regulation of steroid hormone secretion
lightcyan1	GO:2000846	6.31E−04	71.33	0.039	2	8	Regulation of corticosteroid hormone secretion
saddlebrown	GO:0006396	1.11E−05	5.19	3.215	13	678	RNA processing
saddlebrown	GO:0000395	6.59E−05	437.17	0.014	2	3	mRNA 5′-splice site recognition
saddlebrown	GO:0000185	6.06E−04	72.83	0.038	2	8	Activation of MAPKKK activity
saddlebrown	GO:0046886	6.06E−04	72.83	0.038	2	8	Positive regulation of hormone biosynthetic process
saddlebrown	GO:2000271	6.06E−04	72.83	0.038	2	8	Positive regulation of fibroblast apoptotic process
saddlebrown	GO:0060670	7.77E−04	62.42	0.043	2	9	Branching involved in labyrinthine layer morphogenesis
saddlebrown	GO:0008584	8.83E−04	10.4	0.432	4	91	Male gonad development
saddlebrown	GO:0046546	8.83E−04	10.4	0.432	4	91	Development of primary male sexual characteristics
saddlebrown	GO:0045292	9.68E−04	54.61	0.047	2	10	mRNA cis splicing via spliceosome
saddlebrown	GO:0060712	9.68E−04	54.61	0.047	2	10	Spongiotrophoblast layer development

## Discussion

In this study, we utilized the WGCNA to explore the gene co-expression modules networks for expression values of 4,311,721 mRNA in peripheral blood mononuclear cells drawn from MDD, 8 BD, and 24 HC individuals were obtained from GEO (GSE39653) (Savitz et al., 2013). We identified 50 co-expression modules in which the number of eigengenes ranged in size from 42 to 13,240 genes. Two co-expression modules (saddlebrown and lightcyan1) showed striking correlation with the phenotypic trait between MD and healthy controls. Among the 12 genes identified in Savitz et al. as differentially expressed between controls and cases (mood disorder), 6 (IER5, NFKBIZ, CITED2, TNF, SERTAD1, ADM) appeared in module saddlebrown. Based on the GO pathway analysis, biological function of the saddlebrown module and lightcyan1 module were found to be focused on inflammation and neurological response and RNA processing.

The algorithm of WGCNA software could construct a gene co-expression network to provide the expanded explanation of gene expression information. As it has some advantages over traditional approaches to differential expression analysis, the software has been conducted for the gene expression pattern in the mental illness (Geng et al., 2020). WGCNA analysis has been widely used in transcriptional analysis of major depression, schizophrenia, autism, and Alzheimer’s disease (Miller et al., 2008; Voineagu et al., 2011; Ciobanu et al., 2020). Recently, Belzeaux et al. collected a discovery queue and two duplicate queues with similar designs by using WGCNA analysis, 9 of the 59 modules were associated with clinical improvement (Belzeaux et al., 2016). Another study also reported that WGCNA analysis explored candidate modules and central genes associated with subsyndromic depressive symptoms (SSD). Gene expression studies of SSD observed different patterns between cases and controls, which may provide new insights into the molecular mechanisms of SSD (Geng et al., 2020). To the best of our knowledge, this is the first study that used WGCNA to explore candidate modules and hub genes associated with MD.

In the current study, the 6 hub genes (IER5, NFKBIZ, CITED2, TNF, SERTAD1, ADM) appeared in module saddlebrown were among the 12 differentially expressed genes identified in Savitz et al. (2013) This indicates that a significant proportion of differentially expressed genes related to mood disorder may be tightly co-regulated, functionally related, or in the same pathway. IER5, as an immediate early genes/transcription factor, was likely to affect basic cellular functions such as RNA and protein synthesis, neural plasticity, neurotransmission, and metabolism (Cirelli and Tononi, 2000). IER5 gene encodes an activator of HSF1 which was to control hippocampal PSA-NCAM levels through the transcriptional regulation of polysialyltransferases, a process that might be involved in neuronal and behavioral development in mice (Yamano et al., 2020). Transcription of NFKBIZ mediates the transcriptional response to TNF and IL-17A. In fibroblasts, CUX1 and NFKBIZ mediate the synergistic inflammatory response to TNF and IL-17A in stromal fibroblasts (Slowikowski et al., 2020). Moreover, Harrison et al., Inagaki et al., and Savitz et al. showed the correlations between hemodynamic response of the amygdala to sad faces and genes such as CFD and NFKBIZ which are involved in the inflammatory response (Harrison et al., 2009; Inagaki et al., 2012; Savitz et al., 2013). Su et al. reported that the NF-κB was activated in the hippocampi of wild-type (WT) mice after CUMS exposure by regulating the expression of cytokines. Previous studies demonstrated that depression-like behaviors caused by stress were dependent on HMGB1/TLR4/NF-κB and TNF-α/TNFR1/NF-κB signalling pathways in CUMS-exposed mice (Su et al., 2017; Liu et al., 2019; Lu et al., 2019). Arctigenin exerts antidepressant-like effects by attenuating microglial activation and neuroinflammation through the HMGB1/TLR4/NF-κB and TNF-α/TNFR1/NF-κB signalling pathways (Xu et al., 2020). CITED2 represses innate immune cell pathogenic response by modulating broad inflammatory gene programming in macrophages and protecting the host from pathogenic inflammation (Pong Ng et al., 2020). SERTAD1, which appeared to be essential for neuron death in trophic support deprivation *in vitro* and *in vivo* and in models of DNA damage, was associated with Alzheimer’s disease (Biswas et al., 2010). It may therefore be a suitable target for neuropsychiatric diseases, such as MD. Adrenomedullin (ADM) has been confirmed as a vasorelaxant that is part of the first-line protective (i.e., anti-inflammatory) response to toxic or aversive stimuli such as lipopolysaccharide (LPS) (Wong et al., 2005). Genome-wide association study (GWAS) implicated a single nucleotide polymorphism (SNP) in the vicinity of the ADM gene in a sample of subjects with type II BD. In addition, a functional SNP in the ADM gene was associated with response to paroxetine, an SSRI antidepressant (Glubb et al., 2010). Recently, a whole transcriptome RNA-sequencing study revealed 30 genes (included *ADM*) differentially expressed in MDD compared to controls (Mahajan et al., 2018). Together, these data implicate neuro-inflammation in a large number of genes and functional pathways in MD and playing a crucial role in MD. A growing number of studies suggest behavioral and genetic function of the central nervous system, as well as their involvement affected in many neurologic and psychiatric conditions, such as neurodegenerative diseases and mood disorders (Jeremic et al., 2021). This mounting evidence on the involvement of inflammatory/immune systems and their relationships with neurotransmitters seems to represent intriguing avenues for the development of real innovative therapeutic strategies of mood disorders (Mucci et al., 2020). Neuro-inflammation is potentially important in the pathophysiology of MD. Thus, the current study has confirmed the 6 hub genes (IER5, NFKBIZ, CITED2, TNF, SERTAD1, ADM) of neuro-inflammation in MD.

Interestingly, we also found that the GO about the MD was associated with the saddlebrown and lightcyan1 module. Ten of the 19 GO terms pathway in two modules were associated with the steroid hormone secretion pathway, which included negative regulation of steroid hormone secretion (GO:2000832, *p* = 2.29E−05) and regulation of corticosteroid hormone secretion (GO:2000846 *p* = 6.31E−04).

Growing evidence implicates involvement of endogenous glucocorticoids in adverse health effects beyond neurological/neurobehavioral outcomes (neurodegenerative disease, cognitive decline, perceived stress, depression, and suicide) (Thomson et al., 2016). These data provided insight into potential biological mechanisms underlying health impacts and susceptibility in neuropsychiatric diseases, such as MD.

Comparing the results of the WGCNA here with what Savitz et al.’s results were, we think they found that the network of saddlebrown module is clustered based on the similar function of RNA processing. In our study, the RNA processing of mRNA 5′-splice site was recognized, mRNA cis spliced via spliceosome, and branching involved in the labyrinthine layer morphogenesis. Recently, regulating gene expression through splicing, as a novel mechanism, has been described and could contribute to depression by changing gene expression (Le Francois et al., 2018). Alternative splicing is a prevalent modification, especially in human neuronal genes (Kang et al., 2011), resulting in a greater diversity of RNA transcripts (Darnell, 2013; Raj and Blencowe, 2015).

## Conclusion

In this study, we applied WGCNA to transcriptomic data from 21 MDD, 8 BD, and 24 HC individuals that were obtained from GEO (GSE39653). We found a significant overlap for 6 hub genes (ADM, CITED2, IER5, NFKBIZ, SERTAD1, TNF) with similar co-expression and dysregulation patterns associated with mood disorder. Interestingly, we also found that the GO about the MD was associated with the saddlebrown and lightcyan1 modules. These pathways in two modules were associated with the steroid hormone secretion pathway and function of RNA processing, which have been described could contribute to depression. Our findings support other reports on molecular-level immune dysfunction in mood disorder and provide novel insights into the pathophysiology of mood disorder.

## Data Availability

Publicly available datasets were analyzed in this study. This data can be found here: http://www.ncbi.nlm.org/geo/.
