# Supplementary material for: Co-Expression Network Modeling Identifies Specific Inflammation and Neurological Disease-Related Genes mRNA Modules in Mood Disorder
Source: Front Genet. 2022 Mar 21;13:865015. doi: 10.3389/fgene.2022.865015 (PMC8977853; doi:10.3389/fgene.2022.865015)
Supplement: Supplementary file 1 [file DataSheet1.docx]

Table S1 50 unique different colors were identified and represented by WGCNA analysis

| **Module** | **Color** | **Size** | **Module** | **Color** | **Size** |
| --- | --- | --- | --- | --- | --- |
| 0 | grey | 13240 | 26 | darkorange | 117 |
| 1 | turquoise | 3070 | 27 | white | 116 |
| 2 | blue | 1311 | 28 | skyblue | 112 |
| 3 | brown | 897 | 29 | saddlebrown | 109 |
| 4 | yellow | 879 | 30 | steelblue | 107 |
| 5 | green | 810 | 31 | paleturquoise | 103 |
| 6 | red | 723 | 32 | violet | 97 |
| 7 | black | 695 | 33 | darkolivegreen | 92 |
| 8 | pink | 563 | 34 | darkmagenta | 87 |
| 9 | magenta | 531 | 35 | sienna3 | 86 |
| 10 | purple | 313 | 36 | yellowgreen | 85 |
| 11 | greenyellow | 263 | 37 | skyblue3 | 80 |
| 12 | tan | 240 | 38 | plum1 | 76 |
| 13 | salmon | 237 | 39 | orangered4 | 73 |
| 14 | cyan | 236 | 40 | mediumpurple3 | 71 |
| 15 | midnightblue | 217 | 41 | lightsteelblue1 | 71 |
| 16 | lightcyan | 184 | 42 | lightcyan1 | 69 |
| 17 | grey60 | 181 | 43 | ivory | 68 |
| 18 | lightgreen | 173 | 44 | floralwhite | 57 |
| 19 | lightyellow | 166 | 45 | darkorange2 | 54 |
| 20 | royalblue | 160 | 46 | brown4 | 52 |
| 21 | darkred | 148 | 47 | bisque4 | 49 |
| 22 | darkgreen | 144 | 48 | darkslateblue | 49 |
| 23 | darkturquoise | 142 | 49 | plum2 | 48 |
| 24 | darkgrey | 137 | 50 | thistle2 | 42 |
| 25 | orange | 135 |  |  |  |

Table S2 Hub genes from of the saddlebrown module

| **Probe** | **Gene** | **Gene_ID** | **K_within** |
| --- | --- | --- | --- |
| ILMN_1718977_GADD45B | GADD45B | ENSG00000099860 | 1.000 |
| ILMN_3245103_RNU11 | RNU11 | ENSG00000270103 | 0.936 |
| ILMN_3244646_RNU1G2 | RNU1G2 |  | 0.922 |
| ILMN_3236653_RNU1.5 | RNU1.5 |  | 0.916 |
| ILMN_3246273_RNU1.3 | RNU1.3 | ENSG00000207513 | 0.865 |
| ILMN_3245678_RNU1A3 | RNU1A3 |  | 0.839 |
| ILMN_3237617_RNU5A | RNU5A |  | 0.769 |
| ILMN_3240594_RNU4ATAC | RNU4ATAC | ENSG00000264229 | 0.760 |
| ILMN_3309453_RNU4.1 | RNU4.1 |  | 0.751 |
| ILMN_3236713_SNHG1 | SNHG1 | ENSG00000255717 | 0.742 |
| ILMN_3242315_SNORD3D | SNORD3D | ENSG00000199663 | 0.724 |
| ILMN_1721833_IER5 | IER5 | ENSG00000162783 | 0.716 |
| ILMN_3247018_SNORA67 | SNORA67 | ENSG00000252473 | 0.713 |
| ILMN_1780769_TUBB2C | TUBB2C | ENSG00000188229 | 0.663 |
| ILMN_3246433_RNY5 | RNY5 | ENSG00000252310 | 0.638 |
| ILMN_1694327_LOC285176 | LOC285176 |  | 0.630 |
| ILMN_1719695_NFKBIZ | NFKBIZ | ENSG00000144802 | 0.629 |
| ILMN_1750100_TUBB4Q | TUBB4Q | ENSG00000251297 | 0.596 |
| ILMN_3235404_SNORA57 | SNORA57 | ENSG00000223027 | 0.595 |
| ILMN_1663092_CITED2 | CITED2 | ENSG00000164442 | 0.587 |
| ILMN_2096747_SNORA33 | SNORA33 | ENSG00000252045 | 0.586 |
| ILMN_3240220_RNU1F1 | RNU1F1 |  | 0.581 |
| ILMN_3238078_SNORA45 | SNORA45 | ENSG00000212607 | 0.558 |
| ILMN_1728106_TNF | TNF | ENSG00000232810 | 0.537 |
| ILMN_3238554_SNORA80 | SNORA80 | ENSG00000200792 | 0.524 |
| ILMN_3272590_LOC100129975 | LOC100129975 |  | 0.515 |
| ILMN_2085525_SNORA32 | SNORA32 | ENSG00000207503 | 0.514 |
| ILMN_1730773_SNORA70 | SNORA70 | ENSG00000253042 | 0.512 |
| ILMN_3239574_SNORD3A | SNORD3A | ENSG00000263934 | 0.506 |
| ILMN_1794017_SERTAD1 | SERTAD1 | ENSG00000197019 | 0.495 |
| ILMN_1679727_CLK1 | CLK1 | ENSG00000013441 | 0.491 |
| ILMN_2072391_SNORD31 | SNORD31 | ENSG00000201847 | 0.480 |
| ILMN_1689400_CLK1 | CLK1 | ENSG00000013441 | 0.453 |
| ILMN_1673181_SNORD100 | SNORD100 | ENSG00000221500 | 0.452 |
| ILMN_1708934_ADM | ADM |  | 0.115 |

Table S3 The module membership of these 12 genes

| **Module** | **K_within** | **Proble** |
| --- | --- | --- |
| saddlebrown | 0.716 | ILMN_1721833_IER5 |
| saddlebrown | 0.629 | ILMN_1719695_NFKBIZ |
| saddlebrown | 0.587 | ILMN_1663092_CITED2 |
| saddlebrown | 0.537 | ILMN_1728106_TNF |
| saddlebrown | 0.495 | ILMN_1794017_SERTAD1 |
| saddlebrown | 0.115 | ILMN_1708934_ADM |
| lightsteelblue1 | 0.180 | ILMN_1770260_NFKBIZ |
| grey | NA | ILMN_1666269_CTSZ |
| grey60 | 0.371 | ILMN_1742001_CD160 |
| green | 0.436 | ILMN_1777190_CFD |
| darkolivegreen | 0.871 | ILMN_1782305_NR4A2 |
| darkolivegreen | 0.850 | ILMN_2339955_NR4A2 |
| brown4 | 0.219 | ILMN_2320513_APBB3 |
| brown | 0.132 | ILMN_1680692_NUCKS1 |
| black | 0.263 | ILMN_1740772_APBB3 |
